# The Defectiveness of Water When Too Pure

**Published:** 1905-03

**Authors:** 


### THE DEFECTIVENESS OF WATER WHEN TOO PURE.

The admirable natural fitness of the elements for the purposes which nature intended them to serve is forcibly illustrated by certain facts going to show the inferiority, indeed the actual harmfulness, of water as a medium for fresh water animals to be surrounded by when it has been rendered as nearly pure as it is possible to make it. For a number of years there have been before the profession accounts of experiments by competent observers tending to establish as a fact the noxiousness of distilled water to certain crustaceans and other fresh water or amphibious animals. But there has been a weak point in these observations, that of the possibility that the effects noted were not merely those of the purity of the water, but those of its contamination by some poisonous substance in the process of distillation. Mr. G. Bullot, of the Rudolph Spreckels Physiological Laboratory of the University of California (*University of California Publications, Physiology*, vol. i. No. 22) has therefore rendered a tangible service to our knowledge by going over the ground again in a very thorough manner.

Mr. Bullot's experiments were made on fresh-water crustaceans. He finds distilled water highly prejudicial to these animals under all circumstances, but particularly when, as a result of the distillation having been conducted in copper vessels, it contains copper even so minute a proportion as one part in 77,000,000—a fact worth noting in view of the recent proposal to employ copper as an addition to drinking-water for bactericidal purposes. This idea of the noxiousness of copper in such experiments had been suggested before, but none the less Mr. Bullot has done well to investigate it anew. Even from glass vessels water takes up a certain amount of the constituents of that material, but Mr. Bullot shows that it is not to such contamination that the noxiousness of the water is attributable; at least he has distilled water in vessels of quartz and platinum, thereby rendering it presumably free of discoverable foreign matter, and has found it still toxic. It seems justifiable to speak of it as actually toxic, and not merely inadequate to its life-giving functions, for Mr. Bullot finds that the greater the amount of distilled water in which his crustaceans are placed the sooner they die. The bearing of such findings on medicine may not be immediate, but it would be foolhardy to say that they will not at some time be felt. All exact knowledge is desirable, and sooner or later most of it is likely to prove useful.—*New York Medical Journal.*

